# Relationship between clinical parameters and quality of life in primary Sjögren’s Syndrome: a prospective study

**DOI:** 10.1038/s41433-023-02386-2

**Published:** 2023-01-19

**Authors:** E. Greenan, Q. Pilson, J. Ní Gabhann-Dromgoole, C. C. Murphy

**Affiliations:** 1 Royal Victoria Eye and Ear Hospital, Adelaide Rd, Dublin 2, D02 XK51 Ireland; 2 Department of Ophthalmology, RCSI, University of Medicine and Health Sciences, 123 St Stephen’s Green, Dublin 2, D02 YN77 Ireland; 3 School of Pharmacy and Biomolecular Sciences, RCSI, University of Medicine and Health Sciences, 123 St Stephen’s Green, Dublin 2, D02 YN77 Ireland; 4 Department of Ophthalmology, Stepping Hill Hospital, Poplar Grove, Hazel Grove, Stockport, SK2 7JE UK

**Keywords:** Corneal diseases, Pain, Quality of life, Autoimmune diseases

## Abstract

**Objectives:**

To quantify the impact of dry eye disease (DED) on health and vision related quality of life (HR-QOL, VR-QOL) in patients with Primary Sjögren’s Syndrome (pSS).

**Methods:**

Thirty-four participants with a confirmed diagnosis of pSS as per the 2016 ACR EULAR criteria participated. Main outcome measures included ocular surface parameters and HR-QOL and VR-QOL questionnaires. Clinical examination included visual acuity, Schirmer I testing, ocular surface staining (OSS) and measurement of tear film breakup time. The questionnaires included Ocular Surface Disease Index, National Eye Institute Visual Function Questionnaire-25, Short Form-36 (SF-36) and EULAR Sjogren’s Syndrome Patient Reported Index.

**Results:**

Despite the majority of participants (28 female, 6 male, mean age 61.3 years) having attained LogMAR 0.3 or better visual acuity, participants scored low on VR-QOL measures, representing DED related fluctuation in functional vision. All participants suffered from moderate to severe DED. OSS did not correlate with DED symptoms or QOL parameters. Lubricant usage and symptom severity had a statistically moderate to strong negative correlation with VR-QOL and HR-QOL. This was most evident in relation to physical and physiological wellbeing. Compared with normative data, participants had a lower HR-QOL in all scales of the SF-36 ((MD = 9.91 ± 5.16); t(7) = 5.43, *p* = 0.001).

**Conclusions:**

Participants with pSS have a lower perceived QOL especially in relation to physical and mental wellbeing, correlating to severity of DED symptoms and treatment burden. Clinical signs do not align with symptoms. Therefore, clinicians should remain cognisant, adjusting treatment in accordance with patient reported perceptions.

## Introduction

Dry eye disease (DED) is defined as *“a multifactorial disease of the ocular surface characterised by a loss of homeostasis of the tear film, and accompanied by ocular symptoms, in which tear film instability and hyperosmolarity, ocular surface inflammation and damage, and neurosensory abnormalities play aetiological roles”* [1]. Patients affected by DED experience symptoms of discomfort, pain, stinging, grittiness or a foreign body sensation as well as fluctuant vision that can be persistent if the underlying cause is not addressed and managed. Too often, the available treatment options are reactive and fail to provide long-term relief or a cure.

Sjögren’s syndrome (SS) is a chronic autoimmune disease characterised by lymphocytic infiltration, inflammation and ultimately destruction of the lacrimal and salivary glands. This leads to a sicca complex of DED and dry mouth, often accompanied by fatigue and joint pain. Beyond this, 30% to 40% of patients will also go on to develop extra glandular manifestations of the disease [2–4]. This autoimmune exocrinopathy of the lacrimal glands leads to an aqueous deficient tear film and acts as the triggering event to initiate an aggressive and self-perpetuating cycle of DED that has tear film hyperosmolarity and inflammation at its core [5].

Patients carry a significant burden of disease due to DED regardless of the underlying aetiology and overall, it can have a detrimental effect on quality of life for those affected. DED affects patients not only in terms of symptoms endured but also in that of daily functioning, productivity as well as negatively affecting emotional wellbeing [6, 7]. These repercussions have been noted to be more prevalent and severe in patients with pSS [8]. Furthermore, a recent survey of the members of the Sjögren’s Syndrome Foundation revealed that the symptoms of dry eye were the most annoying and activity-limiting aspect of SS [9].

Thus, the purpose of our study was to quantify the effect of DED on the perceived health and vision related quality of life (HR-QOL and VR-QOL) parameters in patients with pSS. We examined the association of HR-QOL and VR-QOL measured using validated QOL tools with DED specific questionnaires and ophthalmic clinical examinations. In doing so, we sought to highlight the reaching impact of DED beyond that of the ocular surface on the QoL of patients with pSS patients.

## Materials and methods

### Participants

Thirty-four participants with a diagnosis of pSS in accordance with the 2016 ACR EULAR diagnostic criteria partook in the study [10]. Written informed consent was obtained prior to participation. Patients with a history of ophthalmic conditions other than refractive error, such as age related macular degeneration or visually significant cataracts were excluded from the study.

### Questionnaires

Four self-administered questionnaires were used to assess VR-QOL and HR-QOL. Questionnaires were mailed to participants in advance of their scheduled clinical assessment along with a cover letter and a study information leaflet. The participants were asked to complete the questionnaires in the week prior to their assessment and to return them to researchers upon attending for ophthalmic examination.

#### Ocular surface disease index (OSDI)

The OSDI is a validated 12-item questionnaire that is used to effectively assess the severity of DED [11]. The self-assessment tool has three subsections; ocular symptoms, vision related function and environmental triggers. A final score out of 100 is calculated, with 0 to 12 representing normal ocular surface, 13 to 22 as mild DED, 23 to 32 as moderate disease and greater than 33 representing severe DED.

#### National eye institute visual function (NEI VFQ-25)

The NEI VFQ-25 is a general questionnaire used to measure the impact of ocular disease on VR-QOL [12]. The questionnaire measures general health and eleven visual function domains; general vision, ocular pain, near and distance vision, vision specific (VS) social functioning, mental health, role difficulties and dependency, driving, colour and peripheral vision. Each subscale is converted into a total score ranging from 0 to 100, with a higher score indicating better VR-QOL.

#### Short form-36 (SF-36)

The SF-36 is a generic measure of perceived HR-QOL in eight domains of daily life. These include physical function, role physical, bodily pain, general health, vitality, social function, role emotional, and mental health. Subscales are calculated ranging from 0 to 100 within each subscale. Higher scores indicated better HR-QOL.

#### EULAR Sjögren’s syndrome patient reposted index (ESSPRI)

The ESSPRI is designed to measure the three main symptoms of pSS; dryness, fatigue, and pain. They are rated on a global assessment scales from 1–10, with a higher score indicating more severe symptoms [13].

### Clinical examination

In each instance, participants underwent a clinical examination by a trained ophthalmologist (authors EG or QP). This included a measure of LogMAR visual acuity and a comprehensive anterior segment evaluation including slit lamp biomicroscopy, evaluation of lid margin, meibomian glands, conjunctiva and corneal surface, along with tests of tear function and grading of the ocular surface status. Tests were performed as described below.

#### Best correct visual acuity (BCVA)

BCVA testing was measured using LogMAR chart. Participants were seated 6 metres from the chart, in standardized low light conditions (approximately 10 cd/m^2^), with their pupils in their natural state.

#### Schirmers I test

Schirmers I test was performed (i.e., without anaesthesia). Participants were asked to look upwards while the sterile filter paper was placed inside the lower lid margin at the junction between the lateral third and medial two-thirds before closing their eyes. After 5 min, the amount of wetting on the filter paper was recorded. A value of less than 5 mm was considered to be severely dry and in keeping with the diagnostic criteria set out by the ACR-EULAR diagnostic criteria [10].

#### Tear break up time (TBUT)

Tear film stability was gauged by instilling a single drop of unpreserved fluorescein hydrochloride 2% into the inferior sac of the conjunctiva on each eye. To reduce variability, participants were asked to blink three to five times before measurements were taken [14]. Cobalt blue light was used for observation. The time taken for the first dry spot to appear after blinking was noted, two measurements were taken and the average recorded [15, 16]. Less than 5 s was recorded as dry [17].

#### Ocular surface diseases grading

Grading of ocular surface disease was performed using two different dyes; unpreserved fluorescein hydrochloride 2% (for cornea only) and lissamine green (for both cornea and conjunctiva). Minims of fluorescein hydrochloride 2% was instilled into the inferior sac of the conjunctiva of each eye and the cobalt blue light of the slit lamp was used for observation. A drop of unit dose saline was instilled on the lissamine green impregnated strips (1.5 mg) and the green dye produced was then instilled into the lacrimal sac. A white light source from the slit lamp was used for visualisation. Using the Oxford grading scheme the corneal surface, temporal and nasal conjunctiva were graded [18]. A total ranging from 0 to 15 score was derived by adding the subscale scores.

### Statistical analysis

Statistical analysis of data was performed using GraphPad Prism 9.0 for Windows (GraphPad Software, La Jolla, CA, USA). Data are expressed as the mean ± standard deviation, and ranges are presented where indicated. Normality of each variable was tested and non-parametric correlation was computed using Spearman r formulation. A correlation factor of <0.3, 0.3–0.5, and 0.5–0.7 was considered to correlate lowly, moderately and strongly, respectively. BCVA and worse clinical parameters (Schirmers I test, TBUT and ocular surface staining) were used in comparative formulations, as they best represented patient’s functional vision and symptoms experienced day to day. SF-36 results were controlled for both age and gender and compared to normative data [19]. Mental and physical component summaries (MCS and PCS) were calculated as per the developers instructions [20]. Differences in response between normative data and that of participants were studied using a paired *t*-test. A *p* < 0.05 was considered statistically significant.

## Results

### Participant characteristics and ocular examinations

Participant demographics and details of ocular examination results are outlined in Table 1. A total of 34 participants, 28 females and 6 males were included in the study. The average age was 61.3 (range 35.7–87.1) years. Of the possible 68 eyes, clinical examination findings were recorded from 67 as one participant had a bandage contact lens in situ for unrelated disease.

**Table 1:** Characteristics of participants, all of which met the 2016 ACR EULAR diagnostic criteria for pSS (*n* = 34).

	Mean	SD	Range	*n*	%
Age (years)	61.3	10.8	35.7–87.1		
Gender					
Male				6	17.6
Female				28	82.4
Immunosuppression					
Hydroxychloroquine				11	32.4
MTX				1	2.9
AZA				1	2.9
Rituximab				1	2.9
Prednisolone				1	2.9
Total				15	44.1
Oral pilocarpine					
				4	11.8
Ocular medications					
Lubricant/day	6.9	5.5	0–20	34	100
ASD				2	5.9
Ciclosporine				2	5.9
Lubricant ointment				7	20.6
BCVA					
Better eye	0.04	0.1	−0.1–0.3		
LogMAR ≤ 0				20	58.8
Worse eye	0.09	0.15	−0.7		
LogMAR ≤ 0				14	41.2
TBUT (sec)					
Worse eye	3.1	1.4	1-Jul	34	100
<5 s				32	91.4
Better eye	*4*	1.8	1-Aug	34	100
<5 s				27	77.1
Oxford staining					
Worse eye	5.4	5.1	0–15	34	100
≥5 +				17	50
Better eye	*3.6*	4.8	0–3.6	34	100
≥5 +				9	34
Schirmer I test					
All participants					
Worse eye	2.2	1.8	0–5	34	100
≤5 mm				34	100
Better eye	3.5	2.4	0–9	34	100
≤5 mm				28	82.4
MGD				22	32.4

*MXT* methotrexate, *AZA* Azathioprine, *ASD* autologous serum drops, *BCVA* Best Correct Visual Acuity, *TBUT* Tear Break Up Time, *MGD* Meibomian gland diseaset.

Twenty participants had a BCVA of LogMAR 0.0 (equivalent to Snellen acuity 20/20) in their better eye (58.8%), and the reminder had a BCVA of better or equal to LogMAR 0.3 (Snellen acuity 20/40). The majority of participants suffered from moderate to severe aqueous deficient dry eye disease combined with evaporative dry eye disease. This is represented by heavy reliance on ocular lubricant usage, (mean 6.9 time per day, range 0–20) and Schirmer I test and TBUT results of 2.2 mm (±1.8) and 3.1 s (±4.1) respectively. The mean Oxford score was 5.4 out of 15, with 17 of the participants eyes scoring ≥ 5 needed for ACR EULAR criteria (50%). Meibomian gland disease was noted in 22 of 67 examined (32.4%).

### VR-QOL and HR-QOL and association with ocular surface parameters

#### OSDI and clinical parameters

Final calculated OSDI scores indicated that the majority of participants suffered with moderate to severe dry eye disease; 6 participants were classified as having scores within normal range (17.6%), 4 had mild disease scores (11.8%), 5 had moderate disease scores (17.6%), and 19 had severe disease scores (55.9%). The mean OSDI symptom score was 36.4 (±24.2), mean OSDI function score was 30.9 (±29.0), mean environment score was 53.8 (±36.7) and the mean OSDI total was 38.6 (±24.2). Table 2 shows the association of the subscale and total OSDI scores with clinical parameters. There was a moderate positive association with statistical significance between BCVA and OSDI function (0.43, *p* = 0.011) and overall scores (0.38, *p* = 0.026). Regarding lubricant usage, there was a strong positive association of significance between frequency of daily use and that of the OSDI subscale of symptoms (0.52, *p* = 0.018), function (0.61, *p* = 0.004) and total OSDI score (0.55, *p* = 0.011). In relation to Schirmer I tests, there was statistically significant positive moderate correlation between its value and OSDI function score (0.36, *p* = 0.028) and had a strong statistically significant positive association with TBUT (0.53, *p* = 0.001). TBUT had a moderate association with OOS (−0.35, *p* = 0.044).

**Table 2:** OSDI subscale and overall score, and heat map representing their correlation with clinical parameters (Spearman correlation).

*OSDI* Ocular Surface Disease Index, *BCVA* Best Corrected Visual Acuity, *Lubricants* daily lubricant usage, *Schirmer* Schirmer I test, *wo* without, *TBUT* Tear Break Up Time, *OSS* Oxford Surface Staining.

*indicated statistical significance (*p* < 0.05).

#### NEI VFQ-25 and clinical parameters

NEI VFQ-25 scores were lowest regarding participant perceived general health (47.6 ± 23.2), vision specific role difficulties (67.8 ± 25.6), ocular pain (71.7 ± 21.4), general vision (72.3 ± 17.4) and vision specific mental health (74.6 ± 17.2).

BCVA held a moderate negative correlation with the majority of the vision related subscales in the NEI VFQ-25, as well as that of ocular pain and VS social functioning. There was a moderate to strong correlation of statistical significance between lubricant usage and VS role difficulties (−0.55, *p* = 0.013) and VS mental health (−0.46, *p* = 0.040). Schirmer results had a moderate positive correlation with VS role difficulties (−0.43, *p* = 0.012). The majority of OSDI subscale scores and overall score showed moderate to strong correlations with NEI VFQ-25 results. These were significant in the instance of OSDI symptoms and general vision (−0.40, *p* = 0.021), ocular pain (−0.34, *p* 0.047), distance activities (−0.37, *p* = 0.032), VS social functioning (−0.45, *p* = 0.007), VS mental health (−0.47, *p* = 0.005), VS dependency (−0.35, *p* = 0.041) and colour vision (−0.48, *p* = 0.003). OSDI function had a significant correlation general vison (−0.43, *p* = 0.011), ocular pain (−0.59, *p* = <0.001), near activities (−0.63, *p* < 0.001), distance activities (−0.53, *p* = 0.001), VS social functioning (−0.50, 0.003), VS mental health (−0.55, *p* 0.001), VS dependency (−0.40, *p* = 0.018), colour vision (−0.50, *p* = 0.003) and peripheral vision (−0.41, *p* = 0.015). In relation to OSDI environment there was again a moderate association with colour vision (−0.36, *p* = 0.039). Total OSDI scores had a modestly negative correlation with that of ocular pain (−0.45, *p* = 0.007), near and distance activities (−0.45, *p* = 0.008; −0.45, *p* = 0.007, respectively), VS social function (−0.50, *p* = 0.003), VS mental health (−0.46, *p* = 0.007), VS dependency (−0.39, *p* = 0.023) and colour vision (−0.52, *p* = 0.002). This data is outlined in full in Table 3.

**Table 3:** NEI VFQ-25 subscale results and heat map representing its correlations against OSDI subscales and clinical parameters (Spearman correlation).

*NEI VFQ-25* National Eye Institute Visual Function Questionnaire-25, *BCVA* Best Corrected Visual Acuity, *Lubricants* daily lubricant usage, *Schirmer* Schirmer I test, *wo* without, *TBUT* Tear Break Up Time, *OSS* Oxford Surface Staining, *VS* Vision Specific.

*indicated statistical significance (*p* < 0.05).

#### SF-36, OSDI and clinical parameters

**Fig. 1:**
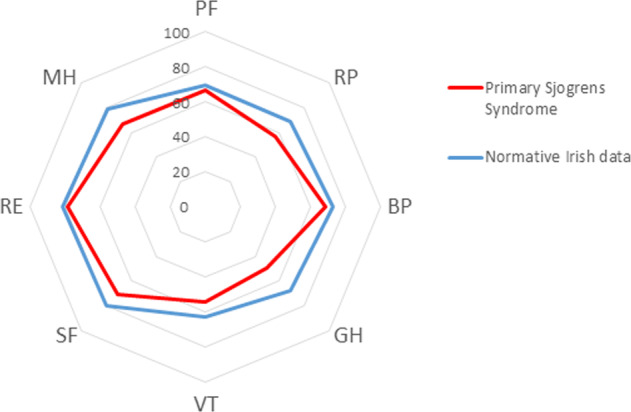
SF-36 scores of normative data and participants with pSS. Results controlled for both gender and age [19]. PF Physical Function, RP Role limitation due to physical health, BP Bodily Pain, GH General Health, VT Vitality, SF Social Function, RE Role limitation due to emotional health, MH Mental Health.

When compared to normative data, participants with pSS had a statistically significantly lower perceived HR-QOL in all scales of the SF-36 (MD = 9.91 ± 5.16); t (7) = 5.43, *p* = 0.001 [19]. This is illustrated in the radar chart in Fig. 1. The mean energy and vitality measured 45.0 (±22.1), role limitations due to physical function 47.7 (±43.5), general health 46.5 (27.4), physical function score 54.9 (±27.8), bodily pain 67.1 (±29.1), mental health 66.5 (±19.9), social function 68.9 (±19.9), role limitations due to emotional health 76.8 (±37.7), and these values were all worse when compared to age and sex matched normative population values for these subscales. The overall mean PCS score was 38.2 (±13.1), indicating a reduced overall physical HR-QOL in comparison to normative data (50.0 ± 10.0). The mean MCS score was lower, but to a lesser degree, than the normative population (46.7, ± 8.4 vs 50.0 ± 10.0).

In relation to subscales and their association with clinical parameters and OSDI score, BCVA had a modest negative associated with role limitation due to physical function (−0.34, *p* = 0.049) and that of emotional function (−0.41, *p* = 0.018). Lubricant use and mental health had a strong, statistically significant negative correlation (−0.62, *p* = 0.003). There was a moderate statistically significant association between Schirmer test results and physical function (−0.38, *p* = 0.032). There was an overall negative correlation between OSDI subscales and SF-36 scores. In relation to symptom score from the OSDI questionnaire, correlations reached a moderate significance in terms of role limitation due to emotional health, mental health and bodily pain (−0.45, *p* = 0.009; −0.39, *p* = 0.023; −0.40, *p* = 0.023). OSDI function moderately correlated significantly with the SF-36 subscales of physical function (−0.38, *p* = 0.031), mental health (−0.41, *p* = 0.017) and the MCS (−0.35, *p* = 0.043). Total OSDI scores were moderately associated with role limitation due to emotional health (−0.37, *p* = 0.036), and mental health (−0.40, *p* = 0.022). These results are outlined in full in Table 4.

**Table 4:** SF-36 subscale scores and heat map representing their correlation with clinical parameters and OSDI score (Spearman correlation).

*PF* Physical Function, *RP* Role limitation due to physical health, *BP* Bodily Pain, *GH* General Health, *VT* Vitality, *SF* Social Function, *RE* Role limitation due to emotional health, *MH* Mental Health, *BCVA* Best Corrected Visual Acuity, *Lubricants* daily lubricant usage, *Schirmer* Schirmer I test, *wo* without, *TBUT* Tear Break Up Time, *OSS* Oxford Surface Staining, *OSDI* Ocular Surface Disease Index.

*indicated statistical significance (*p* < 0.05).

#### ESSPRI OSDI and clinical parameters

ESSPRI scores showed that participants experienced a mean of 6.6 (±1.9), 6.1 (±2.8) and 3.9 (±3.0) in terms of perceived dryness, fatigue and pain respectively, with an overall mean score of 16.6 (±5.6).

Aspects of the ESSPRI had a statistically significant correlation with OSDI scores. Participant’s dryness scores had a moderate statistically significant correlation with BCVA (0.37, *p* = 0.031), OSDI symptoms (0.47, *p* = 0.006), function (0.44, *p* = 0.010) and total OSDI score (0.37, *p* = 0.033). ESSPRI total score correlated moderately with BCVA (0.38, *p* = 0.027) and OSDI symptoms (0.38, *p* = 0.028). Results are outlined fully in Supplementary Information.

## Discussion

This study quantifies perceived HR-QOL and VR-QOL in patients with pSS, highlighting the burden of SS related DED and emphasising the reaching and enduring implications of DED on these patients’ day-to-day life beyond that of clinical signs seen during slit lamp examination.

Recorded Schirmer test and TBUT results had a minimal impact on other clinical and HR-QOL and VR-QOL parameters. However, participants’ measurements were far lower than what is considered within ‘normal range’ creating a mixed picture of DED, both evaporative and aqueous deficient. Those with pSS commonly suffer from both aqueous deficient and evaporative DED due to Meibomian gland dysfunction with gland blockage, drop out and poor quality secretions [21, 22]. Participant’s ocular surface staining score poorly correlated with other clinical parameters, symptoms of DED, HR and VR-QOL measures. Such a discordance between signs and symptoms has previously been documented by other authors and can be attributed to neurosensory abnormalities underpinning DED [1, 23–25]. Consequently, it is important that clinicians are cognisant that clinical signs of DED can poorly correlate with patient symptoms, and thus underestimates their impact on the QOL of patients with DED [26]. Clinicians should weigh and utilise patient reported symptoms and outcomes when judging disease severity and the effectiveness of treatment to ensure the appropriate management of DED. The findings of this study support the wider use of self-administered questionnaires like the SF-36, NEI-VFQ25, ESSPRI and OSDI to measure patient reported outcomes and well-being in clinical practice to assist in therapeutic decision-making. The OSDI is the only one of these questionnaires that is in widespread clinical use.

Participants with pSS in this study reported poor scores in the visual function sections of all questionnaires, and often BCVA was the only objective clinical sign which correlated with QOL measures, accredited to DED related fluctuant vision. Due to DED, patients experienced eye fatigue and blurring of vision throughout the day, as well as discomfort, pain and stinging [27, 28]. Patients struggle to maintain clear vision due to ocular surface damage and tear film instability creating optical aberrations and light scattering along the visual axis resulting in a decrease in contrast sensitivity and degradation of retinal image quality [29–33]. This has a significant limiting effect on patients social and physical functioning, independence in daily life, reflected in our participants NEI-VF 25 results and physical function categories of the SF-36and OSDI as well in as other publications [34, 35]. The effect of fluctuant vision can be far reaching, impacting on mental health, as some studies have postulated that visual disturbance and perceived reduced visual performance can exacerbate patients’ anxiety and depression [36–38].

The participants’ DED played a significant role in their overall perception of SS related desiccation as evidenced by the OSDI correlation with the dryness category of the ESSPRI. Symptoms of DED are persistent, often progressive and can be akin to that of chronic pain syndromes negatively affecting on sleep patterns, mood, cognition and over all mental health [39, 40]. DED has been shown to have a measurable adverse influence on both function and psychological wellbeing of patients, with a higher prevalence of depression and anxiety, which can be especially severe amongst pSS sufferers[8]. Further to this, the severity of DED symptoms and frequency of lubricant drop usage negatively correlated with participant’s emotional wellbeing and mental health components of the SF-36. It has been suggested that frequency of eye drop use may negatively influence psychological wellbeing, those with a higher reliance having an increased likelihood of suffering from anxiety [41].

The impact of SS on HR-QOL was substantial. Overall, the SF-36 results showed that suffering from pSS had a diminishing effect on patients’ perceived HR-QOL when compared to age and gender matched results, evidence replicated in other pSS QOL studies [19, 42–44]. This was most significant in relation to the SF-36 categories of general health, mental health and physical role limitations. In its own right, DED has a corrosive effect on patients QOL, and its impact has been equated to other chronic ailments such as dialysis or moderate to severe angina [45, 46]. Those who also suffer from pSS contend with this as well as complex physical ailments due to extra glandular involvement. This reduces patient’s physical capabilities as well as providing psychological and emotional challenges.

## Conclusion

The symptoms of DED have a significant degrading effect on patient health- and vision-related quality of life in pSS. When managing pSS clinicians should be aware and considerate of the potential mental and physical weight of DED. Furthermore, clinicians should rely more on patient reported outcomes and visual acuity and focus beyond simply assessing traditional clinical signs like TBUT and OSS. The treatment of pSS related DED symptoms should be optimised with the ultimate aim of reducing discomfort and improving patients’ QOL.

## Summary

### What was known before

- In those patients with Primary Sjogren’s Syndrome the symptoms of dry eye disease have a significant degrading effect on health and vision related quality of life.
- Often, the available dry eye disease treatment options for these patients are reactive and fail to provide long-term relief or a cure.

### What this study adds

- When managing Primary Sjogren’s Syndrome clinicians should be aware and considerate of impact of dry eye disease symptoms on patient’s health and vision related quality of life.
- Clinical signs noted on slit lamp examination do not align with symptoms.
- Thus, ophthalmologist should rely more on patient reported outcomes and visual acuity when treating patients with Primary Sjogren’s Syndrome related dry eye disease.
- Treatment should be adjusted in accordance with patient reported perceptions.

## Supplementary information

Supplementary table

## Data Availability

The datasets used and/or analysed during the current study are available from the corresponding author on reasonable request.

## References

[1] Craig JP, Nichols KK, Akpek EK, Caffery B, Dua HS, Joo CK (2017). TFOS DEWS II definition and classification report. Ocul Surf.

[2] Fox RI (2005). Sjogren’s syndrome. Lancet (Lond, Engl).

[3] Yamamoto M, Takeda K, Akira S (2004). TIR domain-containing adaptors define the specificity of TLR signaling. Mol Immunol.

[4] Asmussen K, Andersen V, Bendixen G, Schiodt M, Oxholm P (1996). A new model for classification of disease manifestations in primary Sjogren’s syndrome: evaluation in a retrospective long-term study. J Intern Med.

[5] Bron AJ, de Paiva CS, Chauhan SK, Bonini S, Gabison EE, Jain S (2017). TFOS DEWS II pathophysiology report. Ocul Surf.

[6] Dana R, Meunier J, Markowitz JT, Joseph C, Siffel C (2020). Patient-reported burden of dry eye disease in the United States: results of an online cross-sectional survey. Am J Ophthalmol.

[7] Hossain P, Siffel C, Joseph C, Meunier J, Markowitz JT, Dana R (2021). Patient-reported burden of dry eye disease in the UK: a cross-sectional web-based survey. BMJ Open.

[8] Wan KH, Chen LJ, Young AL (2016). Depression and anxiety in dry eye disease: a systematic review and meta-analysis. Eye (Lond).

[9] Foulks GN, Forstot SL, Donshik PC, Forstot JZ, Goldstein MH, Lemp MA (2015). Clinical guidelines for management of dry eye associated with Sjögren disease. Ocul Surf.

[10] Shiboski CH, Shiboski SC, Seror R, Criswell LA, Labetoulle M, Lietman TM (2017). 2016 American College of Rheumatology/European League Against Rheumatism Classification Criteria for Primary Sjögren’s Syndrome: a consensus and data-driven methodology involving three international patient cohorts. Arthritis Rheumatol.

[11] Schiffman RM, Christianson MD, Jacobsen G, Hirsch JD, Reis BL (2000). Reliability and validity of the Ocular Surface Disease Index. Arch Ophthalmol (Chic, Ill: 1960).

[12] Mangione CM, Lee PP, Gutierrez PR, Spritzer K, Berry S, Hays RD (2001). Development of the 25-item National Eye Institute Visual Function Questionnaire. Arch Ophthalmol (Chic, Ill: 1960).

[13] Seror R, Ravaud P, Mariette X, Bootsma H, Theander E, Hansen A (2011). EULAR Sjogren’s Syndrome Patient Reported Index (ESSPRI): development of a consensus patient index for primary Sjogren’s syndrome. Ann Rheum Dis.

[14] Cho P, Brown B, Chan I, Conway R, Yap M (1992). Reliability of the tear break-up time technique of assessing tear stability and the locations of the tear break-up in Hong Kong Chinese. Optom Vis Sci: Off Publ Am Acad Optom.

[15] Nichols KK, Mitchell GL, Zadnik K (2004). The repeatability of clinical measurements of dry eye. Cornea.

[16] Cho P, Leung L, Lam A, Choi A (1998). Tear break-up time: clinical procedures and their effects. Ophthalmic Physiol Opt J Br Coll Ophthalmic Opt.

[17] Devauchelle-Pensec V, Pennec Y, Morvan J, Pers JO, Daridon C, Jousse-Joulin S (2007). Improvement of Sjogren’s syndrome after two infusions of rituximab (anti-CD20). Arthritis Rheum.

[18] Bron AJ (1997). The Doyne Lecture. Reflections on the tears. Eye (Lond, Engl).

[19] Blake C, Codd MB, O’Meara YM (2000). The Short Form 36 (SF-36) Health Survey: normative data for the Irish population. Ir J Med Sci.

[20] Ware JEKM, Keller SD SF-36 Physical and Mental Health Summary Scales: A User’s Manual Boston, MA. Health Assessment Lab, New England Medical Center. 1994.

[21] Sullivan DA, Dana R, Sullivan RM, Krenzer KL, Sahin A, Arica B (2018). Meibomian gland dysfunction in primary and secondary Sjögren syndrome. Ophthalmic Res.

[22] Zang S, Cui Y, Cui Y, Fei W (2018). Meibomian gland dropout in Sjögren’s syndrome and non-Sjögren’s dry eye patients. Eye.

[23] McMonnies CW (2017). The potential role of neuropathic mechanisms in dry eye syndromes. J Optom.

[24] Belmonte C, Nichols JJ, Cox SM, Brock JA, Begley CG, Bereiter DA (2017). TFOS DEWS II pain and sensation report. Ocul Surf.

[25] Nichols KK, Nichols JJ, Mitchell GL (2004). The lack of association between signs and symptoms in patients with dry eye disease. Cornea.

[26] Friedman NJ (2010). Impact of dry eye disease and treatment on quality of life. Curr Opin Ophthalmol.

[27] Stapleton F, Alves M, Bunya VY, Jalbert I, Lekhanont K, Malet F (2017). TFOS DEWS II epidemiology report. Ocul Surf.

[28] Uchino M, Schaumberg DA (2013). Dry eye disease: impact on quality of life and vision. Curr Ophthalmol Rep.

[29] Koh S (2016). Mechanisms of visual disturbance in dry eye. Cornea.

[30] Koh S, Maeda N, Hirohara Y, Mihashi T, Bessho K, Hori Y (2008). Serial measurements of higher-order aberrations after blinking in patients with dry eye. Investigative Ophthalmol Vis Sci.

[31] Tutt R, Bradley A, Begley C, Thibos LN (2000). Optical and visual impact of tear break-up in human eyes. Invest Ophthalmol Vis Sci.

[32] Goto E, Yagi Y, Matsumoto Y, Tsubota K (2002). Impaired functional visual acuity of dry eye patients. Am J Ophthalmol.

[33] Rolando M, Iester M, Macrí A, Calabria G (1998). Low spatial-contrast sensitivity in dry eyes. Cornea.

[34] Mertzanis P, Abetz L, Rajagopalan K, Espindle D, Chalmers R, Snyder C (2005). The relative burden of dry eye in patients’ lives: comparisons to a U.S. normative sample. Invest Ophthalmol Vis Sci.

[35] Miljanović B, Dana R, Sullivan DA, Schaumberg DA (2007). Impact of dry eye syndrome on vision-related quality of life. Am J Ophthalmol.

[36] Miljanovic B, Dana R, Sullivan DA, Schaumberg DA (2007). Impact of dry eye syndrome on vision-related quality of life. Am J Ophthalmol.

[37] Denoyer A, Rabut G, Baudouin C (2012). Tear film aberration dynamics and vision-related quality of life in patients with dry eye disease. Ophthalmology.

[38] Kawashima M, Uchino M, Yokoi N, Uchino Y, Dogru M, Komuro A (2015). Associations between subjective happiness and dry eye disease: a new perspective from the Osaka study. PLoS One.

[39] Levitt AE, Galor A, Chowdhury AR, Felix ER, Sarantopoulos CD, Zhuang GY (2017). Evidence that dry eye represents a chronic overlapping pain condition. Mol Pain.

[40] Fine PG (2011). Long-term consequences of chronic pain: mounting evidence for pain as a neurological disease and parallels with other chronic disease states. Pain Med (Malden, Mass).

[41] Vakros G, Scollo P, Hodson J, Murray PI, Rauz S (2021). Anxiety and depression in inflammatory eye disease: exploring the potential impact of topical treatment frequency as a putative psychometric item. BMJ Open Ophthalmol.

[42] Stewart CM, Berg KM, Cha S, Reeves WH (2008). Salivary dysfunction and quality of life in Sjögren syndrome: a critical oral-systemic connection. J Am Dent Assoc (1939).

[43] Meijer JM, Meiners PM, Huddleston Slater JJ, Spijkervet FK, Kallenberg CG, Vissink A (2009). Health-related quality of life, employment and disability in patients with Sjogren’s syndrome. Rheumatol (Oxf, Engl).

[44] Dassouki T, Benatti FB, Pinto AJ, Roschel H, Lima FR, Augusto K (2017). Objectively measured physical activity and its influence on physical capacity and clinical parameters in patients with primary Sjögren’s syndrome. Lupus.

[45] Buchholz P, Steeds CS, Stern LS, Wiederkehr DP, Doyle JJ, Katz LM (2006). Utility assessment to measure the impact of dry eye disease. Ocul Surf.

[46] Schiffman RM, Walt JG, Jacobsen G, Doyle JJ, Lebovics G, Sumner W (2003). Utility assessment among patients with dry eye disease. Ophthalmology.

