# Supplementary material for: Relationship between clinical parameters and quality of life in primary Sjögren’s Syndrome: a prospective study
Source: Eye (Lond). 2023 Jan 19;37(13):2685–92. doi: 10.1038/s41433-023-02386-2 (PMC10482965; doi:10.1038/s41433-023-02386-2)
Supplement: Supplementary file 1 — Supplementary table [file 41433_2023_2386_MOESM1_ESM.docx]

Table 1: ESSPRI scores and heat map representing their correlation with clinical parameters and OSDI score (Spearman correlation).

| **ESSPRI Subscale** | | **Mean (SD)** | **BCVA** | **Lubricants** | **Schirmer** | **TBUT** | **OSS** | **OSDI Symptoms** | **OSDI Function** | **OSDI Environment** | **OSDI Total** | |
| --- | --- | --- | --- | --- | --- | --- | --- | --- | --- | --- | --- | --- |
| **Dryness** | | 6.6 (1.9) | 0.37* | 0.23 | -0.14 | -0.29 | -0.05 | 0.47* | 0.44* | 0.21 | 0.37* | |
| **Fatigue** | | 6.1 (2.8) | 0.34 | 0.00 | 0.04 | -0.13 | 0.06 | 0.22 | 0.18 | 0.11 | 0.17 | |
| **Pain** | | 3.9 (3.0) | 0.19 | -0.07 | 0.18 | 0.17 | -0.06 | 0.25 | 0.21 | 0.18 | 0.24 | |
| **Total** | | 16.6 (5.6) | 0.38* | 0.04 | 0.14 | -0.04 | -0.03 | 0.38* | 0.32 | 0.21 | 0.31 | |
| -1 | **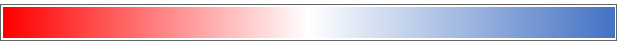** | | | | | | | | | | | +1 |

*ESSPRI; EULAR Sjögren’s Syndrome Patient Reported Index BCVA; Best Corrected Visual Acuity, Lubricants; daily lubricant usage, Schirmer; Schirmer I test, wo; without, TBUT; Tear Break Up Time, OSS; Oxford Surface Staining , OSDI; Ocular Surface Disease Index. * indicated statistical significance (p<0.05).*
